# The MOnitoring Resynchronization dEvices and CARdiac patiEnts (MORE-CARE) Randomized Controlled Trial: Phase 1 Results on Dynamics of Early Intervention With Remote Monitoring

**DOI:** 10.2196/jmir.2608

**Published:** 2013-08-21

**Authors:** Giuseppe Boriani, Antoine Da Costa, Renato Pietro Ricci, Aurelio Quesada, Stefano Favale, Saverio Iacopino, Francesco Romeo, Arnaldo Risi, Lorenza Mangoni di S Stefano, Xavier Navarro, Mauro Biffi, Massimo Santini, Haran Burri, On Behalf Of The MORE-CARE Investigators

**Affiliations:** ^1^Institute of CardiologyDepartment of Experimental, Diagnostic and Specialty MedicineUniversity of Bologna, S Orsola-Malpighi University HospitalBolognaItaly; ^2^University HospitalSt EtienneFrance; ^3^San Filippo Neri HospitalRomeItaly; ^4^University General HospitalValenciaSpain; ^5^University HospitalBariItaly; ^6^Anthea HospitalBariItaly; ^7^Fondazione Policlinico Tor Vergata HospitalRomeItaly; ^8^Medtronic Clinical Research InstituteEMEA Regional Clinical CenterRomeItaly; ^9^Medtronic IbéricaScientific & Clinical DepartmentMadridSpain; ^10^Cardiology Service, University Hospital of GenevaGenevaSwitzerland; ^11^see Multimedia Appendix 3 for collaborators

**Keywords:** cardiac resynchronization therapy, heart failure, alerts, remote monitoring, telemedicine

## Abstract

**Background:**

Remote monitoring (RM) in patients with advanced heart failure and cardiac resynchronization therapy defibrillators (CRT-D) may reduce delays in clinical decisions by transmitting automatic alerts. However, this strategy has never been tested specifically in this patient population, with alerts for lung fluid overload, and in a European setting.

**Objective:**

The main objective of Phase 1 (presented here) is to evaluate if RM strategy is able to reduce time from device-detected events to clinical decisions.

**Methods:**

In this multicenter randomized controlled trial, patients with moderate to severe heart failure implanted with CRT-D devices were randomized to a Remote group (with remote follow-up and wireless automatic alerts) or to a Control group (with standard follow-up without alerts). The primary endpoint of Phase 1 was the delay between an alert event and clinical decisions related to the event in the first 154 enrolled patients followed for 1 year.

**Results:**

The median delay from device-detected events to clinical decisions was considerably shorter in the Remote group compared to the Control group: 2 (25^th^-75^th^ percentile, 1-4) days vs 29 (25^th^-75^th^ percentile, 3-51) days respectively, *P=*.004. In-hospital visits were reduced in the Remote group (2.0 visits/patient/year vs 3.2 visits/patient/year in the Control group, 37.5% relative reduction, *P*<.001). Automatic alerts were successfully transmitted in 93% of events occurring outside the hospital in the Remote group. The annual rate of all-cause hospitalizations per patient did not differ between the two groups (*P=*.65).

**Conclusions:**

RM in CRT-D patients with advanced heart failure allows physicians to promptly react to clinically relevant automatic alerts and significantly reduces the burden of in-hospital visits.

**Trial Registration:**

Clinicaltrials.gov NCT00885677; http://clinicaltrials.gov/show/NCT00885677 (Archived by WebCite at http://www.webcitation.org/6IkcCJ7NF).

## Introduction

Modern cardiac resynchronization therapy defibrillators (CRT-D) are equipped with reliable diagnostics able to provide a series of alerts, including lung fluid accumulation [1], occurrence of atrial fibrillation (AF) [2], or technical issues. Early diagnosis and intervention may play a crucial role in minimizing major cardiovascular events and reducing hospitalization. Several major device manufacturers offer remote monitoring (RM) capabilities [3,4] with the aim of reducing regular follow-up visits [5] and unnecessary interim visits, or of dealing with the more complex perspective of disease management [6]. RM allows physicians to remotely access patient data and to be notified of clinical events by means of the automatic transmission of alert messages. Previous trials such as TRUST [7] and CONNECT [8] have shown that RM is safe and reduces delay in detection of events such as AF. However, these trials either excluded [7] or included only a minority of patients with biventricular defibrillators (CRT-D) [8] and did not include an alert on lung fluid accumulation, which is potentially useful in the context of heart failure management. In addition, the aforementioned trials [7,8] as well as the EVOLVO trial [9] were not strictly focused on NYHA III-IV heart failure patients, a setting where reduction of morbidity and access to hospitals may have a great significance for both the patient and the health care system.

MORE-CARE is a multicenter randomized trial conducted in Europe and designed in two phases [10]. Phase 1 was aimed at evaluating whether RM of CRT-D patients could shorten the time from onset of a clinically relevant event to clinical decisions in comparison with standard management (scheduled in-office visits only). The second phase of MORE-CARE is currently ongoing and is targeted at assessing whether clinical decision making guided by RM exerts a positive impact on patient outcome (death from any cause, cardiovascular and device-related hospitalization) in comparison to standard care [10]. This paper presents the results of Phase 1 of the study.

## Methods

### Remote Monitoring With CareLink Network

**Figure 1:**
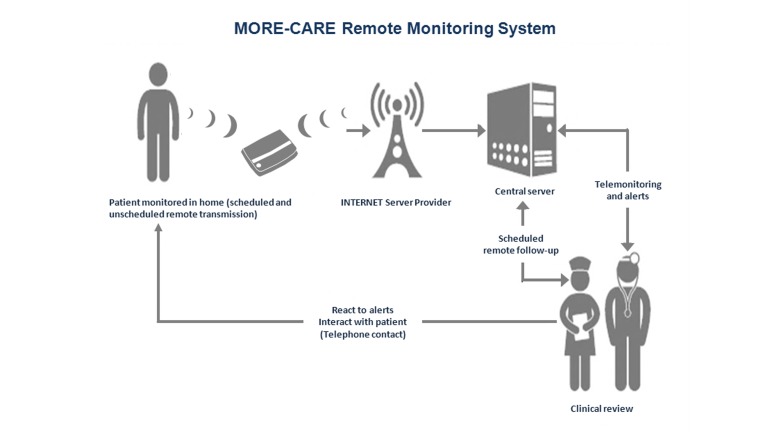
Remote monitoring system platform and interactions between health care professionals and patient.

CareLink Network, as detailed in Multimedia Appendix 1, is a platform for remote monitoring of implantable cardiac devices, which consists of implantable devices provided with wireless telemetry, CareLink monitor (CLM), and the CareLink website (CW). The system allows patients to send comprehensive implant device data to their clinic from home or any location where CareLink is available and that is equipped with an analog telephone line or cellular connection. The organization of the platform and how patients and health care professionals interact are shown in Figure 1. More specifically, every communication between implantable cardiac devices and CLM is based on the Conexus wireless telemetry technology, which uses the Medical Implant Communications Service (MICS) radio frequency band (between 402 and 405 MHz), specifically designed for medical devices and targeted to reduce the risk of interfering with other users of the same band. In case of either scheduled or device-detected event transmission, device information are collected by CLM using the aforementioned wireless communication system and then transmitted via a private data network by means of a phone line connection. Unique credentials created at the time of manufacture and stored in each monitor are used to authenticate the monitor to the CareLink network. Health care providers can analyze the transmitted data via the Internet by using a Web browser to access the CW. The site is also used to enroll clinic users and patients and perform other administrative duties. The Medtronic CareLink network operates on the Windows operating system with database support based on Microsoft’s SQL Server software.

With regard to data protection, health care professionals access patient data via the Internet through a connection to a Microsoft Internet Information Server (IIS). In addition, every user session is protected by means of the 128-bit Secure Sockets Layer (SSL) encryption system.

### Study Design

**Figure 2:**
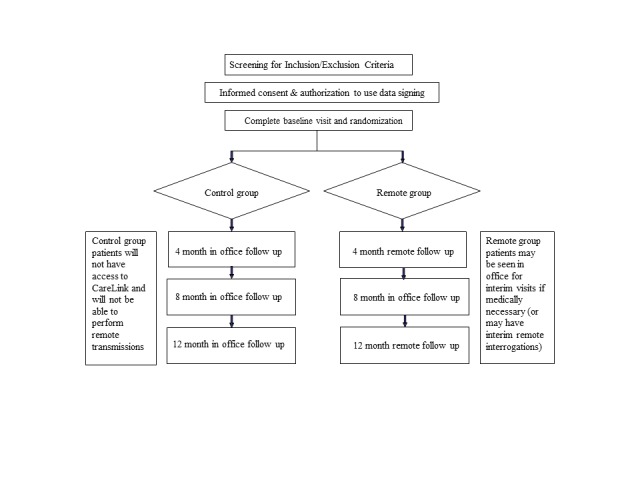
Scheduling of follow-up in the Remote group (with RM and in-office follow-up) and in the Control group (with in-office follow-up only).

The MORE-CARE study is an international, prospective, multicenter randomized controlled trial in patients with a Medtronic CRT-D, designed to compare disease management guided by RM with the CareLink network with standard clinical practice. The trial design has been reported in detail elsewhere [10], and the flow chart is shown in Figure 2. In summary, patients in sinus rhythm with de novo implantation of CRT-D for systolic heart failure with NYHA class III/IV (and a left ventricular ejection fraction (LVEF) <35% were randomized 1:1 to wireless RM Remote group or to a Control group. Patients in the Remote group had in-office visits at baseline and at 8 months, and remote follow-ups performed at 4 and 12 months with activation of automatic alerts (for AF, lung fluid via OptiVol monitoring, device integrity, ineffective shocks, or inactivated ventricular fibrillation (VF) detection/therapy). Control group patients had in-office visits performed at baseline and at every 4 months. Audible alerts for device integrity issues or for inactivated VF detection/therapy were activated in both groups. The institutional ethics committees approved the protocol at all 32 centers involved. All patients were enrolled after signing an informed consent form.

### Programming of Diagnostic Features

In the Remote group, automatic RM alerts for lung fluid accumulation (using OptiVol fluid status monitoring), atrial tachyarrhythmia (AT/AF), lead and device integrity, or inactivated VF detection/therapy were turned on at baseline using standardized predefined thresholds, which could later be modified at the physicians’ discretion. All audible alerts were disabled with the exception of those related to lead and device integrity and programming.

Due to technological aspects of the RM system, any device-detected event for AT/AF, fast ventricular rate during AT/AF and shock triggers a remote notification of the episode and automatically disables the corresponding alert, which may be re-enabled only by means of an in-office device check (alert re-arming). For patients in the Control group, only audible alerts for system integrity and programming issues were enabled. Tachycardia detection and treatment were performed according to the standard practice of the individual centers, as well as optimization of atrio-ventricular (AV) and ventricular-ventricular (VV) intervals for CRT (see Multimedia Appendix 2).

### Objectives

The primary endpoint of Phase 1 (whose participants are reported in (see Multimedia Appendix 3) was to demonstrate a reduction of the time from onset of an actionable device-detected event to a clinical decision. A clinical decision was defined by at least one of the following: change in drug therapy, device reprogramming, patient education (specific advice on salt, fluid check, exercise, behavior, etc), as well as planning of hospital admission for other interventions (eg, electrical cardioversion, radiofrequency ablation, etc). For appropriate clinical decisions and patient management, physicians were aided by specific flowcharts suggesting clinical actions for each type of alert (system performance alerts, device shocks, AF “rhythm control” strategy, AF “rate control” strategy, and OptiVol events). The flowcharts are shown in Multimedia Appendices 4-8. The time of a device-detected event was defined as the day on which the criterion for an alert was satisfied (for subjects in the Control group, this was taken into account as well, even though the alert was switched off). If no actions were taken following the acknowledgement of a device event, it was not considered for primary endpoint adjudication.

The primary endpoint was defined as the delay between onset of the actionable device-detected event to a clinical decision related to that event. Endpoints were validated by a blinded Endpoint Adjudication Committee (see Multimedia Appendix 3). Phase 1 included as secondary objectives an exploratory analysis of the impact of RM on quality of life (by means of the Minnesota Living with Heart Failure Questionnaire) and clinical status (measured by the Clinical Composite Score).

### Data Collection

Phase 1 of the MORE-CARE study required the collection of clinical and device data at scheduled and unscheduled visits (either ambulatory or remotely performed). The onset day of all device-detected events in the Control group patients was determined by evaluating the device memory information collected at each in-office visit. For the Remote group patients, dates of alert transmissions and dates when alerts were reviewed by either nurses or physicians were collected by each study center; the date of alert transmission was considered as the onset day of a device-detected event. In addition, all clinical actions by physicians on the basis of either device memory information or device alert notification were collected and dated; the clinical actions date to treat a specific event was considered as the end date of such event and used to determine the delay between the event onset to a clinical decision. Therefore, for each event, 3 different times have been collected: (1) time of event detection (start time), (2) time to when the physician was able to review the event (time of event revision), and (3) time to clinical actions taken to treat the event (time to clinical decision).

### Statistical Analysis

In accordance with the sample size estimation reported in the study design [10], the sample size requirement was 77 subjects per study arm. In the current report of Phase 1 results, data analysis was performed according to the intention-to-treat principle. The analysis for the primary endpoint includes all randomized subjects who experienced at least one event. Similar to a previous study [8], if a patient experienced multiple events of a specific type (eg, ≥6 h AT/AF burdens in a day) between 2 consecutive evaluations, only the first of these was paired with the next device interrogation/visit and counted toward the analysis. For every patient, an average time from event onset to clinical decision was calculated for each type of event and entered in the analysis per event type.

Descriptive statistics are reported as mean ± 1 standard deviation for normally distributed continuous variables, or medians with 25^th^-75^th^ percentiles for skewed variables. Normality of distribution was tested by the Kolmogorov-Smirnov test. Absolute and relative frequencies are reported for categorical variables. Continuous Gaussian variables were compared by the Student’s *t* test for independent samples, while skewed distributions were compared using the Mann-Whitney nonparametric test. Differences in proportions were compared by applying Chi-square analysis. Rates of events were computed per 100 person years, as number of occurred events out of patient exposure time and reported separately for each arm. The exposure time was computed from the date of randomization to the date of the last available information for each patient, either dropped out or died. Rates were compared by means of the Comparison Incidence Rates (Large Sample) Test. An alpha-level of .05 was considered for each test. All statistical analyses were performed by using SAS 9.3 version software.

## Results

### Study Population

**Figure 3:**
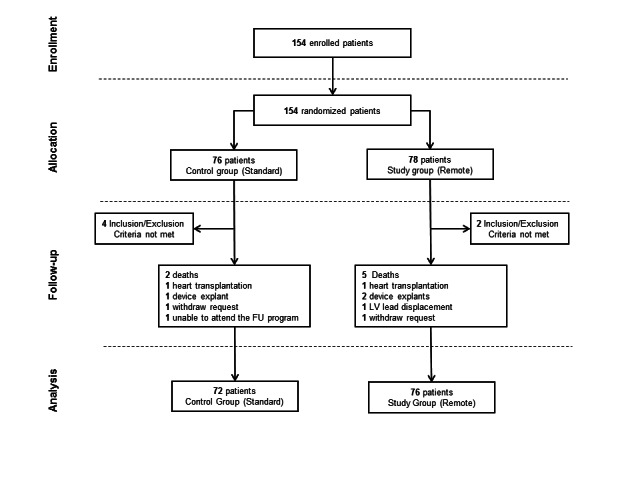
Phase 1 follow-up experience flow-chart.

A total of 154 patients were enrolled from May 2009 through April 2010 from 32 centers in 6 different countries (France, Hungary, Israel, Italy, Spain, and Switzerland). The final patient cohort object of analysis comprised 148 patients (76 in the Remote group and 72 in the Control group see Figure 3). Demographic data and clinical parameters of the population under analysis were similar between the study arms at the time of enrollment (Table 1). The median follow-up duration was 12.0 months (25^th^-75^th^ percentile, 11.8-12.6 months) with 1709 cumulative months of follow-up.

### Device-Detected Events and Remote Transmission

Of the 148 patients, 105 (71%) experienced at least 1 event satisfying the criteria for triggering a device alert: 57 patients (75%) in the Remote group and 48 patients (67%) in the Control group (*P=*.28). There were 166 alerts in the Remote group and 114 episodes matching the alert criteria in the Control group. OptiVol threshold crossing and AT/AF burden were the most frequent events (Table 2). In particular, the observed rate of OptiVol events was similar between the 2 arms (1.6 events/year in the Remote group, 1.5 events/year in the Control group, *P=*.59), while the rate of events of AT/AF burden and fast ventricular rate during AF episodes was higher in the Remote group (0.7 events/year) compared to the Control arm (0.2 events/year, *P*<.001).

Of the 166 alerts in the Remote group, 144 (87%) were successfully transmitted. The remaining 22 alerts (13 %) in the Remote group were not successfully transmitted because the patient was admitted to hospital before transmission in 11 cases, the monitor was not set up in 8 cases, there were connection problems with the phone line in 2 cases, and the patient was not at home in 1 case. Discounting the alerts with failed transmission due to hospital admission, successful transmission occurred in 144/155 (93%) of events.

For the 166 alerts in the Remote group, the median delay between triggering of the alert to when the event was reviewed (remotely or in-office) was 3 days (25^th^-75^th^ percentile, 1-10 days) compared with the median time of 37 days (25^th^-75^th^ percentile, 14-71 days) for the 114 device events of the Control group (*P*<.001, Table 2).

### Time From Actionable Device-Detected Event to Clinical Decisions

**Figure 4:**
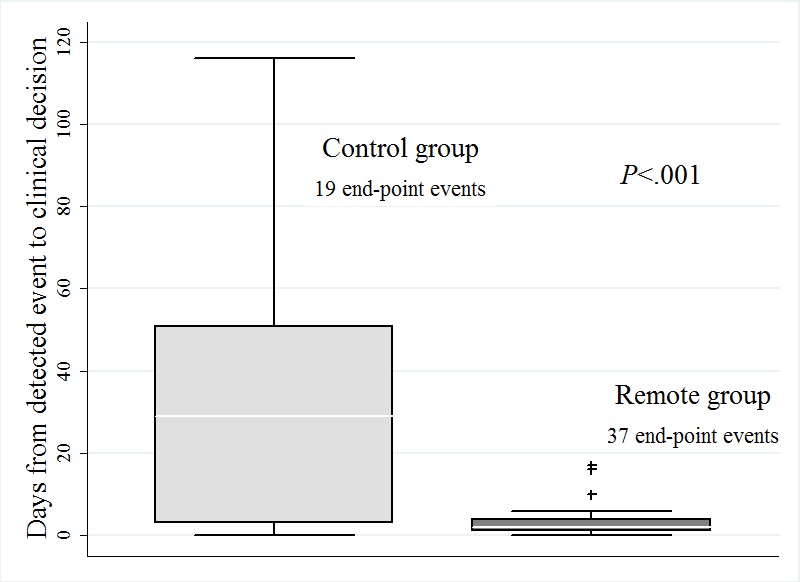
Time from device events to clinical decisions for the phase 1 primary end-points (19 in Control group and 37 in Remote group); box-and-whisker plots show the quartiles with the medians labeled, and the whiskers extended to the lower and the upper adjacent value; plus symbols show the outside values.

Overall, 56 device-detected events led to at least one clinical decision taken by a physician participating in the study protocol (37 events in 23 Remote group patients and 19 events in 15 Control group patients). Device-detected episodes of lung fluid accumulation (53%, n=30) and AT/AF burden above the pre-specified limit (34%, n=19) were the most frequent events leading to consequent clinical actions (Table 3). The median time from the event onset to related clinical decisions was significantly shorter in the Remote group vs the Control group (2 days vs 29 days, *P=*.004, 93% relative reduction, Figure 4).

**Figure 5:**
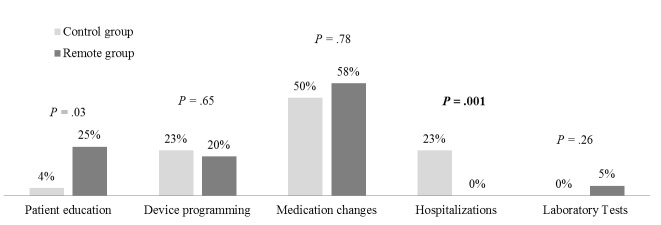
Distribution of specific clinical actions related to device-detected events in the Remote group (n=43) and in the Control group (n=26) respectively.

Of all 56 clinical decisions consequent to device-detected events, 44 of them involved 1 clinical action, 11 had 2 clinical actions and only 1 case had 3 clinical actions performed at the same time (Table 4). Therefore, a total of 69 clinical actions (43 in the Remote group and 26 in the Control group) were taken on the basis of the above-mentioned 56 device-detected events. In both groups, the majority of clinical actions were medication changes (58% in the Remote group and 50% in the Control group, *P*=.78). Device re-programming constituted 20% of clinical actions in the Remote group and 23% in the Control group (*P=*.65), while hospitalizations were decided only for 6 patients in the Control group and none of the patients in the Remote group (*P=*.001, Figure 5).

### In-hospital Visits

**Figure 6:**
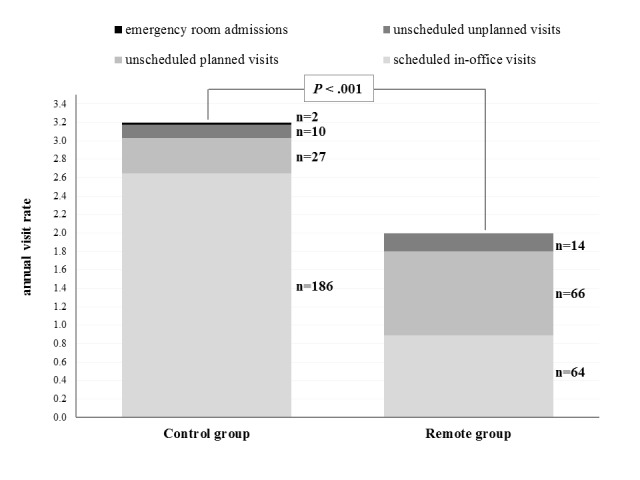
Annual rates per randomization group of scheduled visits (in-office visits performed as per protocol requirement), unscheduled planned (in-office visits not required by the protocol, not patient initiated), and unplanned visits (in-office visits not required by the protocol, patient initiated) and emergency room admissions (for each randomization group and for each type of visit, the total number of occurrences is displayed beside the corresponding bar).

There were a total of 375 scheduled follow-ups: 189 for the 76 Remote group patients (125 remote follow-ups and 64 in-office visits) and 186 in-office visits for the 72 patients of the Control group. Overall, taking into account both scheduled and unscheduled visits (in a referral clinic) plus emergency department visits (with or without subsequent hospitalization) a 37.5% reduced burden was observed in the Remote group (144 visits, corresponding to 2.00 visits/year vs 225 visits, corresponding to 3.20 visits/year in the Control group, *P*<.001, Figure 6).

### Hospital Admissions

During the follow-up of Phase 1, there were 19 hospitalizations for various causes (related to 18 patients) in the Remote group and 22 hospitalizations (related to 16 patients) in the Control group (*P=*.65).

### Quality of Life and Clinical Status

The patient’s quality of life was assessed by means of the Minnesota Living with Heart Failure Questionnaire. Baseline values were comparable between the Remote group (41; 25^th^-75^th^ percentile, 16-62) and the Control group (40; 25^th^-75^th^ percentile, 18-53, *P=*.38). The change in quality of life from baseline to the 8^th^ month was not different for the Remote group (-17; 25^th^-75^th^ percentile, -32 to -2) compared to the Control group (-10; 25^th^-75^th^ percentile, -23 to 0, *P=*.45). The change in clinical status during the trial from enrollment to the 12-month follow-up was similar in both groups according to the Clinical Composite Score. In the Remote group, 54% of patients were defined as “improved”, 35% as “unchanged”, and 11% as “worsened”, while in the Control group these values were 48%, 38%, and 14% respectively (*P=*.69).

### Deaths and Study Exits

During the course of Phase 1, 7 patients died for the following reasons: heart failure (3 patients in the Remote group and 1 patient in the Control group), complications after aortic surgery (1 patient in the Remote group), stroke (1 patient in the Control group), and chronic kidney disease (1 patient in the Remote group). Furthermore, 9 patients exited prematurely from the trial for reasons reported in Figure 3.

**Table 1:** Demographics and baseline clinical parameters.

Subject characteristics		Control group, n=72	Remote group, n=76	*P* value
Male gender, n (%)		54 (75.0)	55 (72.4)	.72
Age, years		68±9	67±10	.63
Ischemic heart disease, n (%)		32 (44.4)	39 (51.3)	.40
**NYHA functional classification at implant, n (%)**				.59
	Class III	70 (98.5)	70 (94.5)	
	Class IV	1 (1.4)	4 (5.4)	
Previous myocardial infarction, n (%)		30 (41.7)	39 (51.3)	.24
Hypertension, n (%)		27 (37.5)	30 (39.5)	.81
History of coronary artery intervention, n (%)		21 (29.2)	22 (29.0)	.98
History of valvular surgery, n (%)		8 (11.1)	5 (6.6)	.33
Chronic Obstructive Pulmonary Disease (COPD), n (%)		15 (20.8)	13 (17.0)	.56
AF paroxysmal, n (%)		8 (11.1)	15 (19.7)	.15
AF persistent, n (%)		1 (1.4)	4 (5.3)	.37
Implant for primary prevention, n (%)		65 (90.3)	68 (89.5)	.87
Diabetes, n (%)		23 (32.4)	26 (35.6)	.68
QRS (ms)		148±30	155±25	.11
LVEF (%)		27±7	27±6	.69
ACE inhibitor or ARB, n (%)		60 (83.3)	64 (84.2)	.89
ß-blocker, n (%)		63 (87.5)	66 (86.8)	.91
Diuretic, n (%)		69 (95.8)	71 (93.4)	.52
Antiarrhythmic agents, n (%)		17 (23.6)	18 (23.7)	.99

**Table 2:** Device-detected events—delays from a device-detected event to review of the alert (including alerts with unsuccessful transmissions in the Remote group, which were evaluated during an in-office visit).

Device-detected event	Total # of device-detected events (# of patients)		# of days from device-detected event to event reviewing Median (25^th^-75^th^ percentile)		*P* value
	Control group	Remote group	Control group	Remote group	
Lead impedances out of range	5 (4)	2(1)	6 (0-22)	12 (0-23)	1.000
VF detection/therapy off	0 (0)	3 (2)	-	0 (0-0)	-
AT/AF burden: at least 6 hours of AT/AF in a single day	9 (7)	39 (12)	51 (5-59)	2 (1-7)	.002
Fast V rate during AT/AF: Mean V rate of at least 100 bpm a day with at least 6 hrs of AT/AF	5 (4)	7 (3)	57 (2-68)	1 (0-6)	.19
Number of shocks delivered in an episode (at least two)	1 (1)	5 (5)	0 (0-0)	0 (0-1)	.73
OptiVol threshold crossing for lung fluid accumulation	94 (47)	110 (55)	39 (22-72)	4 (1-12)	<.001
Total	114 (48)	166 (57)	37 (14-71)	3 (1-10)	<.001

**Table 3:** Delay in clinical decisions—delays from a device-detected event to a clinical decision.

Device-detected event	# of actionable device-detected events followed by a clinical decision (# of patients)		# of days from actionable device-detected event to clinical decision Median (25^th^-75^th^ percentile)		*P* value
	Control group	Remote group	Control group	Remote group	
Lead impedances out of range	2 (2)	0 (0)	24 (13-35)	-	-
VF detection/therapy off	0 (0)	0 (0)	-	-	-
AT/AF burden: at least 6 hrs of AT/AF in a single day	5 (5)	14 (7)	51 (0-59)	2 (1-3)	.51
Fast V rate during AT/AF: Mean V rate of at least 100 bpm a day with at least 6 hrs of AT/AF	0 (0)	2 (2)	-	9 (1-17)	-
Number of shocks delivered in an episode (at least two)	1 (1)	2 (2)	0 (0-0)	0 (0-0)	.50
OptiVol threshold crossing for lung fluid accumulation	11 (9)	19 (15)	29 (9-31)	3 (1-6)	.002
Total	19 (15)	37 (23)	29 (3-51)	2 (1-4)	.004

**Table 4:** Distribution of clinical actions among all actionable device-detected events (19 in the Control group and 37 in the Remote group) followed by a clinical decision.

Actionable device-detected event	Patient education		Device programming			Medication changes		Hospitalizations		Laboratory tests	
	Cont. group	Rem. group	Cont. group	Rem. group		Cont. group	Rem. group	Cont. group	Rem. group	Cont. group	Rem. group
AT/AF burden: at least 6 hrs of AT/AF in a single day (Control group n=5, Remote group n=14)	6	4	2		2	4	8	2	0	0	1
Fast V rate during AT/AF: Mean V rate of at least 100 bpm a day with at least 6 hrs of AT/AF (Control group n=0, Remote group n=2)	0	0	0	0		0	1	0	0	0	1
# of shocks delivered in an episode (at least 2) (Control group n=1, Remote group n=2)	0	0	0	2		0	1	1	0	0	0
OptiVol threshold crossing for lung fluid accumulation (Control group n=11, Remote group n=19)	1	6	3	4		9	13	2	0	0	0
Lead impedances out of range (Control group n=2, Remote group n=0)	0	0	1	0		0	0	1	0	0	0

## Discussion

### Key Findings

The MORE-CARE trial is the first European, large-scale randomized study evaluating disease management guided by RM (including lung fluid overload alerts) in a population comprised exclusively of CRT-D patients with advanced heart failure. The main finding of Phase 1 of the study that we report here is that a wireless RM strategy permits physicians to take clinical decisions 27 days sooner as compared to standard in-office care, while reducing the total number of in-hospital visits.

### Comparison With Other Trials

Several recent studies [7-9,11-14] have demonstrated the benefits associated with remote patient monitoring in terms of early detection of relevant events as well as reduction of in-office follow-up visits. However, none of these studies was dedicated specifically to CRT-D patients with advanced heart failure, and most were conducted in the United States. Recently, the results of the EVOLVO trial were reported, showing a significant reduction in emergency visits in patients on RM [9]. The EVOLVO trial, however, differed significantly from MORE-CARE in that the patient population was enrolled exclusively in one region of Italy, with a mixture of ICD and CRT-D patients of whom >80% had NYHA class I/II heart failure, with activation of audible alerts in the control arm, and without evaluation of delay in medical decisions [9,15].

The Phase 1 results of MORE-CARE revealed a median 27-days reduction in delay from actionable event detection to medical decision for the Remote group compared to the Control group. This reduction was even greater than the median 17-days reduction observed in the CONNECT trial, most probably due to different in-office follow-up intervals (3 months vs 4 months respectively). The delay in reviewing events was considerably shorter in the Remote arm. For AF burden for example, this delay was reduced by a median of 49 days, which compared favorably to the median of 34 days observed in the TRUST study (probably also in part due to differences in follow-up intervals). Delay from the AF alert to clinical action was thereby significantly reduced (2 vs 51 days). This is particularly important in the case of new AF episodes because timely diagnosis and prompt treatment may minimize thromboembolic complications [16-19] and may prevent heart failure (HF) decompensation and inappropriate shocks. In the EVOLVO study, the overall reduction in delay to reviewing of alerts was 23 days, compared to 34 days in MORE-CARE [10]. The difference may be explained by activated audible alerts in the EVOLVO control group. Audible alerts may indeed be useful for device integrity issues but may lead to increased rates of hospital visits or admissions for OptiVol alerts, as shown in the DOT-HF study [15,20]. These data underline the importance of RM with automatic wireless alerts notified to physicians, rather than monitoring based on audible alerts delivered to patients, as a strategy for avoiding unnecessary hospital visits and use of resources.

In addition to timely reviewing of alerts and clinical decision making, the prerequisite for improved patient outcome resulting from RM is the reliable transmission of these alerts. The CONNECT trial showed that 45% of all alerts were not transmitted, mainly due to the monitor not being set up [8]. The percentage of unsuccessful alert transmissions in MORE-CARE was considerably less, 13% in total or only 7% if failed transmission due to hospital admission is discounted. Patient education and logistics for implementing RM may have partly accounted for these differences.

In our study, the number of detected AF events was higher in the Remote group compared to the Control group. This can be explained by taking into account the higher percentage of paroxysmal AF at baseline in the former group compared to the latter and by the fact that alerts are re-armed by in-office device interrogations. Therefore, a single episode of persistent AF may generate multiple alerts if the patient undergoes several in-office device interrogations that are prompted by the alerts.

Monitoring of HF decompensation with OptiVol has been evaluated in several trials [20-23], none of which involved RM. The CONNECT trial did not include the use of the OptiVol algorithm as the feature is not available as an alert in the United States. As reported by a previous study [1], intrathoracic impedance appears to be inversely correlated with pulmonary capillary wedge pressure and its decrease may serve as an early notification to identify patients at high risk of impending exacerbation of congestive heart failure [21,22]. The PARTNERS-HF study [23] has shown that integrating different diagnostics (eg, presence of arrhythmias, patient activity, heart rate variability, and nocturnal heart rate) significantly improved the ability to identify patients at risk of heart failure events, beyond the use of intrathoracic impedance alone. In addition to reliable alert transmission and prompt response to these alerts, appropriate interpretation of diagnostics is likely to affect the ability of an RM strategy to improve patient outcome. We included specific clinical pathways in the MORE CARE trial to favor appropriate alert management. Following analysis of device diagnostics and patient phone contact (specified in the clinical pathway), only a minority (17%) of the OptiVol alerts resulted in clinical action.

Our results showed a significant reduction in the rate of in-office visits in the Remote group compared to the Control group, despite an increased number of unscheduled visits resulting from alerts, which is in line with previous trials that did not specifically target patients with CRT-D [7-9]. This suggests a potential benefit of RM in terms of health care logistics and costs in this patient population with advanced heart failure. However, a more thorough analysis, taking into account all costs of in-office and remote activities, is needed to confirm this aspect.

Even though not significant, there was a trend in greater improvement in quality of life at 8 months in the Remote group compared to the Control group. It is likely that the effect of CRT (rather than follow-up strategy) may be preponderant in improving quality of life early after CRT implantation. Other endpoints such as hospitalizations and mortality were not significantly different between the groups.

### Limitations

Phase 1 of MORE-CARE was not powered for evaluating the impact of RM on cardiovascular and device-related hospitalizations and mortality, which are being studied in the second phase of the trial. There were only a few cases of system integrity alerts because of the limited 1-year follow-up. These aspects will be better evaluated with the longer observation period of the ongoing trial.

### Conclusion

The Phase 1 results of the MORE-CARE randomized study indicate that RM allows a significant reduction in delay from event onset to clinical decisions. In spite of a reduced number of in-hospital visits compared to patients with standard follow-up, we found no significant differences among the groups in terms of quality of life and clinical status. The impact of RM on clinical aspects of disease management in heart failure patients needs to be assessed in the second phase of the trial. Finally, these findings are reported for the first time in a European setting, in a study cohort consisting entirely of CRT-D patients with advanced heart failure in whom remote disease management included alerts for lung fluid overload.

## Multimedia Appendix 1

Carelink system description and general workflow for Carelink scheduled and alert transmission.

## Multimedia Appendix 2

Implantable cardiac device concepts.

## Multimedia Appendix 3

List of Steering Committee members, Endpoint Adjudication Committee members, investigators (and centers) participating in the MORE-CARE Phase 1.

## Multimedia Appendix 4

Flowchart for managing system performance alerts.

## Multimedia Appendix 5

Flowchart for atrial fibrillation episodes. “Rhythm control” strategy.

## Multimedia Appendix 6

Flowchart for atrial fibrillation episodes. “Rate control” strategy.

## Multimedia Appendix 7

Flowchart for managing the possible fluid accumulation alerts (Optivol).

## Multimedia Appendix 8

Flowchart for managing device shock and ICD therapies alerts.

## Multimedia Appendix 9

CONSORT-EHEALTH checklist V1.6.2 [24].

## Abbreviations

ACE: angiotensin-converting enzyme
ARB: angiotensin receptor blocker
AF: atrial fibrillation
AT: atrial tachycardia
AV: atrio-ventricular
CLM: CareLink monitor
COPD: chronic obstructive pulmonary disease
CRT-D: cardiac resynchronization therapy defibrillators
CW: CareLink website
HF: heart failure
LVEF: left ventricular ejection fraction
MICS: Medical Implant Communications Service
NYHA: New York Heart Association
OptiVol: fluid status monitoring tool
QRS: combination of three of the graphical deflections seen on a typical electrocardiogram
RM: remote monitoring
VF: ventricular fibrillation
V rate: ventricular rate
VT: ventricular tachycardia
VV: ventricular-ventricular
