# Supplementary material for: The MOnitoring Resynchronization dEvices and CARdiac patiEnts (MORE-CARE) Randomized Controlled Trial: Phase 1 Results on Dynamics of Early Intervention With Remote Monitoring
Source: J Med Internet Res. 2013 Aug 21;15(8):e167. doi: 10.2196/jmir.2608 (PMC3758044; doi:10.2196/jmir.2608)
Supplement: Supplementary file 1 [file jmir_v15i8e167_app1.pdf]

# Multimedia Appendix 2

# The MOnitoring Resynchronization dEvices and CARdiac patiEnts (MORE-CARE) Randomized Controlled trial: phase 1 results on early decision making with remote monitoring

Giuseppe Boriani, MD PhD[a], Antoine Da Costa, MD[b], Renato Pietro Ricci, MD[c], Aurelio Quesada, MD[d], Stefano Favale, MD[e], Saverio Iacopino, MD[f], Francesco Romeo, MD[g], Arnaldo Risi PhD[h], Lorenza Mangoni di S. Stefano, MS[h], Xavier Navarro, MD[i], Mauro Biffi, MD[a], Massimo Santini, MD[c], Haran Burri, MD[j] on behalf of MORE-CARE Investigators *****

From the [a]Institute of Cardiology, University of Bologna, Bologna, Italy; [b]University Hospital, St. Etienne, France; [c]San Filippo Neri Hospital, Rome, Italy; [d]University General Hospital,Valencia, Spain; [e]University Hospital, Bari, Italy; [f]Anthea Hospital, Bari, Italy; [g]Fondazione Policlinico Tor Vergata Hospital, Rome, Italy, [h] Clinical Department Medtronic Rome, Italy, [i]Scientific & Clinical Department Medtronic Ibérica, Madrid, Spain, [j]University Hospital, Geneva, Switzerland.

**CARELINK SYSTEM.**

Medtronic Carelink Network is the system used in the MORE-CARE trial to perform the remote monitoring (RM) strategy in heart failure (HF) patients treated with implantable cardiac resynchronization defibrillators (CRT-D).

The Carelink Network system is comprised of the following components :

- Cardiac implantable devices. Devices used in the trial were equipped with diagnostic tools able to monitor and send alerts transmission for device related events (lead impedances out of range, lead integrity, low battery, etc…), arrhythmia related events (ventricular fibrillation detecion, atrial tachycardia or atrial fibrillation, fast ventricular rate during episodes of AF, shocks, etc…) and lung fluid accumulation (Optivol algorithm).
- Carelink Monitor (CLM). The CLM is a telemetry device designed for use primarily in the patient's home. CLM uses a radio frequency telemetry system for wireless communication with the patient's implanted device. After receiving all device data, CLM transmits information to an internet server provider.
- Carelink Website (CW). Once transmitted, device data can be viewed by physicians as well as by allowed clinical personnel through a secure Internet website.

*Carelink transmission.*

Device data can be transmitted from patient's home to CW by using either manual or automatic transmissions. A manual transmission is activated by patient who directly allows the CLM to receive device data which should be sent to CW. Carelink automatic transmissions are divided into two groups: scheduled and alert notifications.

A scheduled transmission is comprised of the following steps (Figure 1):

1. Physician may set-up scheduled transmissions using the CW;
2. At a scheduled date the implanted device will automatically send data to CLM via wireless connection;
3. CLM will automatically send device data using a specific bandwidth to a remote server;
4. Physician can review transmitted data via Internet by accessing the CW.

An alert notification consists of the following steps (Figure 2):

1. In case of detection of relevant clinical events or device-related problems, data will be trasmitted to CLM via wireless connection;
2. The CLM will automatically send device data using a specific bandwidth to a remote server;
3. Physician receives the alert notification (via email, SMS, etc…);
4. Physician, after having reviewed the transmitted data through CW, can contact the patient in order to assess the health status and/or to schedule an in-office visit.

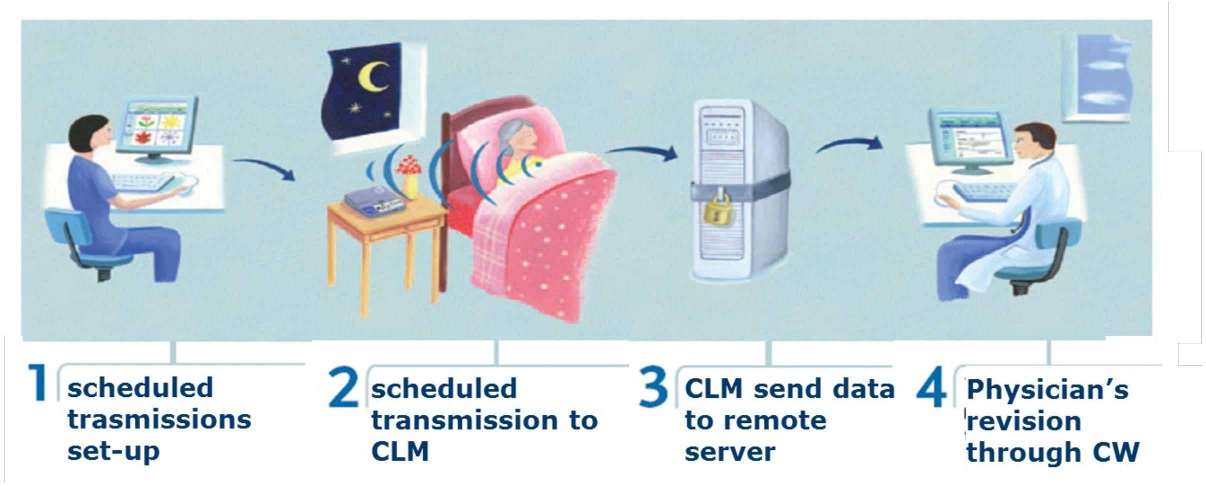


**Figure 1**. General work-flow for Carelink scheduled transmission.

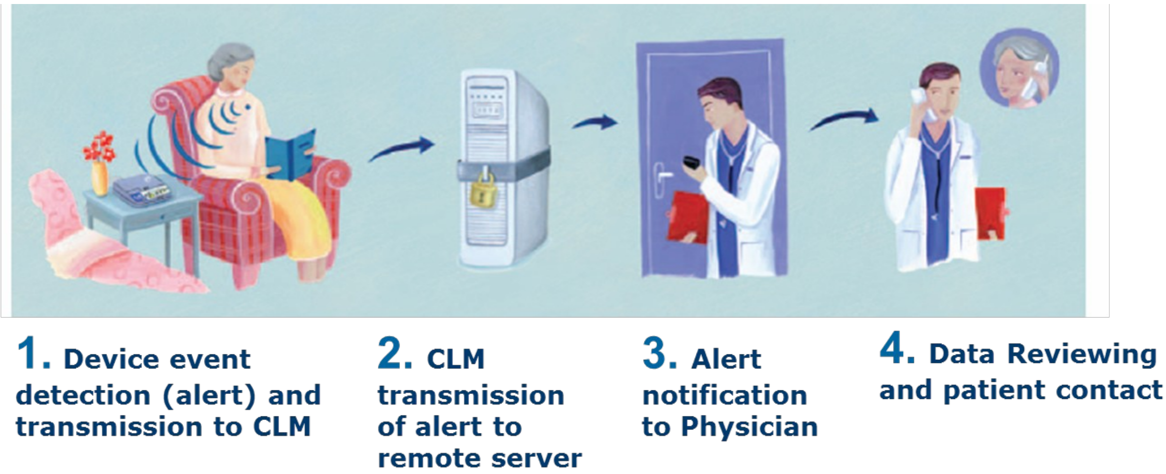


**Figure 2**. General work-flow for alert transmission.
