# Supplementary material for: The MOnitoring Resynchronization dEvices and CARdiac patiEnts (MORE-CARE) Randomized Controlled Trial: Phase 1 Results on Dynamics of Early Intervention With Remote Monitoring
Source: J Med Internet Res. 2013 Aug 21;15(8):e167. doi: 10.2196/jmir.2608 (PMC3758044; doi:10.2196/jmir.2608)
Supplement: Supplementary file 2 [file jmir_v15i8e167_app2.pdf]

Multimedia Appendix 3

The MOnitoring Resynchronization dEvices and CARdiac patiEnts (MORE-CARE) Randomized Controlled trial: phase 1 results on early decision making with remote monitoring

Giuseppe Boriani, MD PhD[a], Antoine Da Costa, MD[b], Renato Pietro Ricci, MD[c], Aurelio Quesada, MD[d], Stefano Favale, MD[e], Saverio Iacopino, MD[f], Francesco Romeo, MD[g], Arnaldo Risi PhD[h], Lorenza Mangoni di S. Stefano, MS[h], Xavier Navarro, MD[i], Mauro Biffi, MD[a], Massimo Santini, MD[c], Haran Burri, MD[j] on behalf of MORE-CARE Investigators *****

From the [a]Institute of Cardiology, University of Bologna, Bologna, Italy; [b]University Hospital, St. Etienne, France; [c]San Filippo Neri Hospital, Rome, Italy; [d]University General Hospital,Valencia, Spain; [e]University Hospital, Bari, Italy; [f]Anthea Hospital, Bari, Italy; [g]Fondazione Policlinico Tor Vergata Hospital, Rome, Italy, [h]Clinical Department Medtronic Rome, Italy, [i]Scientific & Clinical Department Medtronic Ibérica, Madrid, Spain, [j]University Hospital, Geneva, Switzerland.

IMPLANTABLE CARDIAC DEVICE CONCEPTS

Audible alerts.

Most of Medtronic implantable cardiac devices, as those used in the MORE-CARE trial, are equipped with diagnostic tools able to monitor and detect the occurrence of specific events, such as atrial tachycardia or atrial fibrillation, fast ventricular rate during episodes of atrial fibrillation, system integrity issues, and lung fluid accumulation. In addition, those devices offer the possibility to notify the occurrence of aforementioned events by means of alert transmissions and/or audible alerts.

Alert transmissions are allowed only for patients previously provided with a Carelink Network system. Differently from alert transmissions, audible alerts can be programmed without any specific restriction and consists of an acoustic alert emitted by implantable device in case of event detection.

AV and VV intervals for CRT.

Modern CRT-D devices are equipped with the capability to program the atrio-ventricular delay (i.e. time between an atrial polarization and the next ventricular polarization) as well as the interventricular delay (polarization of right and left ventricular) ; atrio-ventricular and interventricular delay are usually referred as AV and VV intervals respectively. The opportunity to manage AV and VV intervals in CRT-D devices allows physician to set-up these parameters in order to restore the coordination of contraction and relaxation among cardiac chambers, leading to improved exercise tolerance, cardiac remodeling (reduction in left ventricular [LV] volumes and improvement in LV ejection fraction), and a better survival in HF patients. The procedure to determine the best choice for the AV and VV intervals (optimization of AV and VV intervals) is usually accomplished by means of echocardiographic assessment.
