# Supplementary material for: The MOnitoring Resynchronization dEvices and CARdiac patiEnts (MORE-CARE) Randomized Controlled Trial: Phase 1 Results on Dynamics of Early Intervention With Remote Monitoring
Source: J Med Internet Res. 2013 Aug 21;15(8):e167. doi: 10.2196/jmir.2608 (PMC3758044; doi:10.2196/jmir.2608)
Supplement: Supplementary file 3 [file jmir_v15i8e167_app3.pdf]

The MOnitoring Resynchronization dEvices and CARdiac patiEnts (MORE-CARE) Randomized Controlled trial: phase 1 results on early decision making with remote monitoring

Giuseppe Boriani, MD PhD[a], Antoine Da Costa, MD[b], Renato Pietro Ricci, MD[c], Aurelio Quesada, MD[d], Stefano Favale, MD[e], Saverio Iacopino, MD[f], Francesco Romeo, MD[g], Arnaldo Risi PhD[h], Lorenza Mangoni di S. Stefano, MS[h], Xavier Navarro, MD[i], Mauro Biffi, MD[a], Massimo Santini, MD[c], Haran Burri, MD[j] on behalf of MORE-CARE Investigators *

From the [a]Institute of Cardiology, University of Bologna, Bologna, Italy; [b]University Hospital, St. Etienne, France; [c]San Filippo Neri Hospital, Rome, Italy; [d]University General Hospital,Valencia, Spain; [e]University Hospital, Bari, Italy; [f]Anthea Hospital, Bari, Italy; [g]Fondazione Policlinico Tor Vergata Hospital, Rome, Italy, [h] Clinical Department Medtronic Rome, Italy, [i]Scientific & Clinical Department Medtronic Ibérica, Madrid, Spain, [j]University Hospital, Geneva, Switzerland.

Steering Committee: Boriani G (Institute of Cardiology, University of Bologna, Bologna, Italy), Burri H (University Hospital, Geneva, Switzerland), Da Costa A (University Hospital, St. Etienne, France), Favale S (University Hospital, Bari, Italy), Kautzner J (IKEM, Prague, Czech Republic), Navarro X (Scientific & Clinical Department Medtronic), Quesada A (University General Hospital, Valencia, Spain), Ricci RP (San Filippo Neri Hospital, Rome, Italy).

End-point Adjudication Committee: Militianu A (Camel Medical Center, Haifa, Israel), Oliva F (Niguarda Ca'Granda Hospital, Milan, Italy), Perego GB (Istituto Auxologico Italiano IRCCS, Milan, Italy).

Investigators (and centers) participating in the MORE-CARE phase 1: Ammann P (Kantonsspital St.Gallen, St. Gallen, Switzerland), Angotti A (Ospedale Pugliese e Ciaccio, Catanzaro, Italy), Babuty D (CHRU, Tours, France), Belvito C (Cliniche Gavazzeni, Bergamo, Italy), Benko O (Wolfson Medical Center, Holon, Israel), Barany T (Semmelweis University AOK, Budapest, Hungary), Bertaglia M (Ospedale Civile, Mirano, Venice, Italy), Biffi M (Institute of Cardiology, University of Bologna, Bologna, Italy), Bizeau O (Hopital La Source, Orleans, France), Bordachar P (Hôpital Cardiologique du Haut Lévêque, Bordeaux, France), Borrello F (Ospedale Pugliese e Ciaccio, Catanzaro, Italy), Boriani G (Institute of Cardiology, University of Bologna, Bologna, Italy), Burri H (University Hospital, Geneva, Switzerland), Bucher U (Kantonsspital St.Gallen, St. Gallen, Switzerland), Calvi V (Ospedale V.E. Ferrarotto, Catania, Italy), Cano O (Hospital Universitario La Fé, Valencia, Spain), Casolo G (Ospedale Unico della Versilia, Lido di Camaiore (LU), Italy), Chevalier P (Hopital Louis Pradel, Lyon, France), Da Costa A (University Hospital, St. Etienne, France), Davy JM (Hopital Arnaud de Villeneuve, Montpellier, France), Diemberger I (Institute of Cardiology, University of Bologna, Bologna, Italy), Di Grazia A (Ospedale V.E. Ferrarotto, Catania, Italy), Dugo D (Ospedale V.E. Ferrarotto, Catania, Italy), Favale S (University Hospital, Bari, Italy), Fedele F (Policlinico Umberto I, Rome, Italy), Forleo G (Policlinico Tor Vergata, Rome, Italy), Franciosa P (Policlinico Umberto I, Rome, Italy), Frey C (Stadtspital Triemli, Zürich, Switzerland), Giunta G (Policlinico Umberto I, Rome, Italy), Geist M (Wolfson Medical Center, Holon, Israel), Geller L (Semmelweis University AOK, Budapest, Hungary), Gemperle M (Kantonsspital St.Gallen, St. Gallen, Switzerland), Georger F (CHU, Narbonne, France), Halfin Z (Wolfson Medical Center, Holon, Israel), Gioffrè Fabrizio (Ospedale San Paolo, Milan, Italy), Iacopino S (Sant'Anna

Hospital, Catanzaro, Italy), Jiménez J (University General Hospital, Valencia, Spain), Lazarus A (Clinique Bizet, Paris, France), Lombardi F (Ospedale San Paolo, Milan, Italy), Leal del Ojo J (Hospital Universitario de Valme, Sevilla, Spain), Lilli A (Ospedale Unico della Versilia, Lido di Camaiore (LU), Italy), Lucca E (Cliniche Gavazzeni, Bergamo, Italy), Maglia G (Ospedale Pugliese e Ciaccio, Catanzaro, Italy), Mariani M (Ospedale San Salvatore, Pesaro, Italy), Martignani C (Institute of Cardiology, University of Bologna, Bologna, Italy), Mascioli G (Cliniche Gavazzeni, Bergamo, Italy), Menachemi D (Wolfson Medical Center, Holon, Israel), Menicagli B (Istituto di Fisiologia Clinica - CNR, Pisa, Italy), Merkely B (Semmelweis University AOK, Budapest, Hungary), Mihi Natividad (University General Hospital, Valencia, Spain), Milhem A (Centre Hospitalier, La Rochelle, France), Morichelli L (San Filippo Neri, Rome, Italy), Müller A (Stadtspital Triemli, Zürich, Switzerland), Olague J (Hospital Universitario La Fé, Valencia, Spain), Osca J (Hospital Universitario La Fé, Valencia, Spain), Osztheimer I (Semmelweis University AOK, Budapest, Hungary), Panchetti L (Istituto di Fisiologia Clinica - CNR, Pisa, Italy), Piacenti M (Istituto di Fisiologia Clinica - CNR, Pisa, Italy), Pierantozzi A (Ospedale San Salvatore, Pesaro, Italy), Pirola A (University General Hospital, Valencia, Spain), Pisapia A (Hopital Saint Joseph, Marseille, France), Quesada Dorador A (University General Hospital,Valencia, Spain), Ragusa A (Ospedale V.E. Ferrarotto, Catania, Italy), Romeo F (Policlinico Tor Vergata, Rome, Italy), Rossi A (Istituto di Fisiologia Clinica - CNR, Pisa, Italy), Sancho Tello J (Hospital Universitario La Fé, Valencia, Spain), Santini L (Policlinico Tor Vergata, Rome, Italy), Santini M (San Filippo Neri Hospital, Rome, Italy), Schillaci V (Ospedale V.E. Ferrarotto, Catania, Italy), Startari U (Istituto di Fisiologia Clinica - CNR, Pisa, Italy), Sticherling C (Universitätsspital, Basel, Switzerland), Stuber T (Triemli Hospital, Zurich, Switzerland), Stettler C (University Hospital, Geneva, Switzerland), Sunthorn H (University Hospital, Geneva, Switzerland), Svetlich C (Ospedale Unico della Versilia, Lido di Camaiore (LU), Italy), Tahin T (Semmelweis University AOK, Budapest, Hungary), Tarchitzky DB (Wolfson Medical Center, Holon, Israel), Tarricone D (Ospedale San Paolo, Milan, Italy), Tarsi G (Ospedale San Salvatore, Pesaro, Italy), Testarmata P (Ospedale San Salvatore, Pesaro, Italy), Zbinder R (Stadtspital Triemli, Zürich, Switzerland), Ziacchi M (Institute of Cardiology, University of Bologna, Bologna, Italy), Zima E (Semmelweis University AOK, Budapest, Hungary).
