# Supplementary figures and images for: The MOnitoring Resynchronization dEvices and CARdiac patiEnts (MORE-CARE) Randomized Controlled Trial: Phase 1 Results on Dynamics of Early Intervention With Remote Monitoring
Source: J Med Internet Res. 2013 Aug 21;15(8):e167. doi: 10.2196/jmir.2608 (PMC3758044; doi:10.2196/jmir.2608)

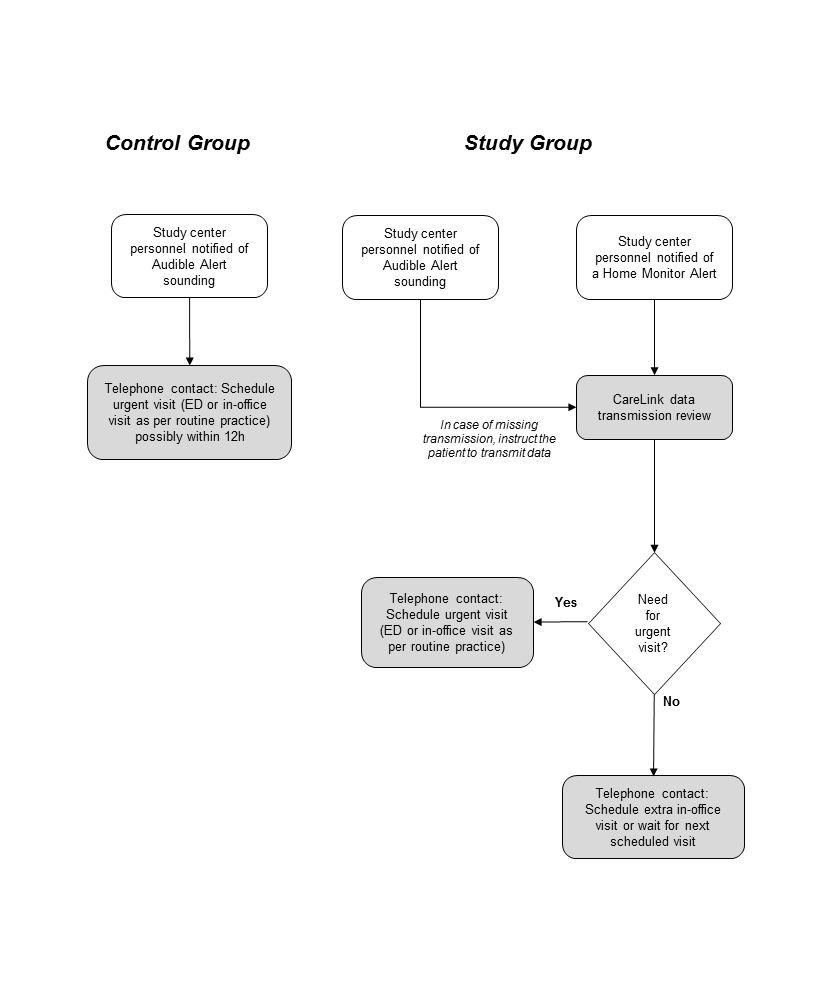

Supplement: Supplementary file 4 [file jmir_v15i8e167_app4.png]

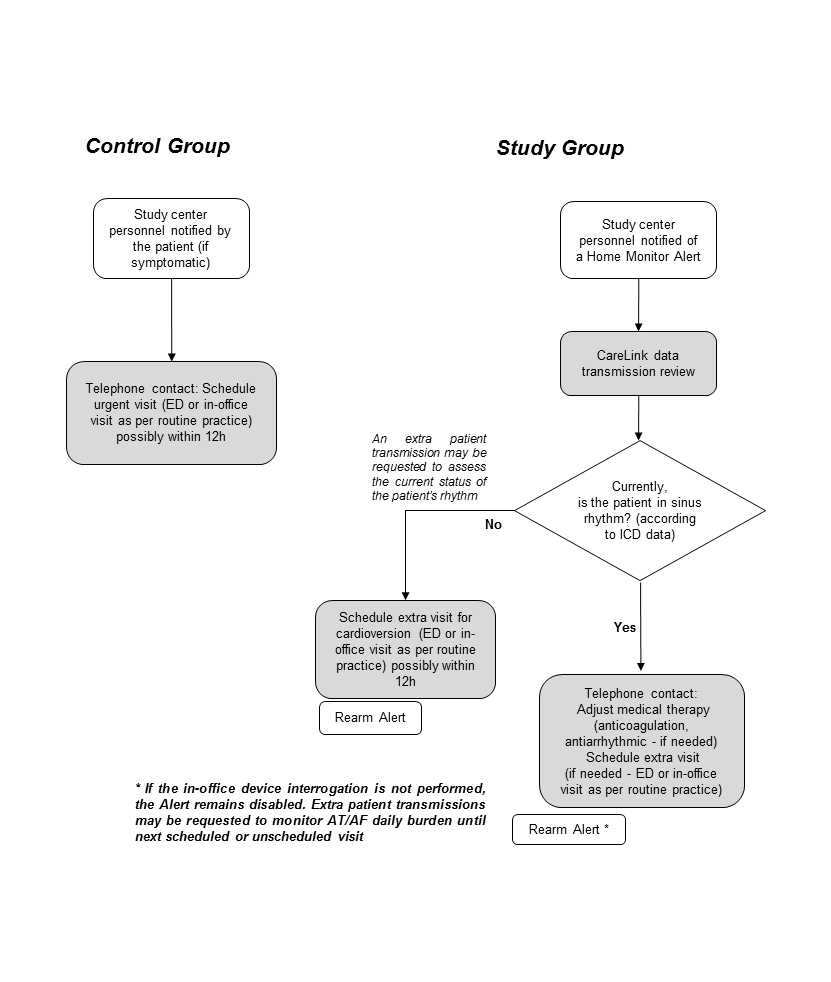

Supplement: Supplementary file 5 [file jmir_v15i8e167_app5.png]

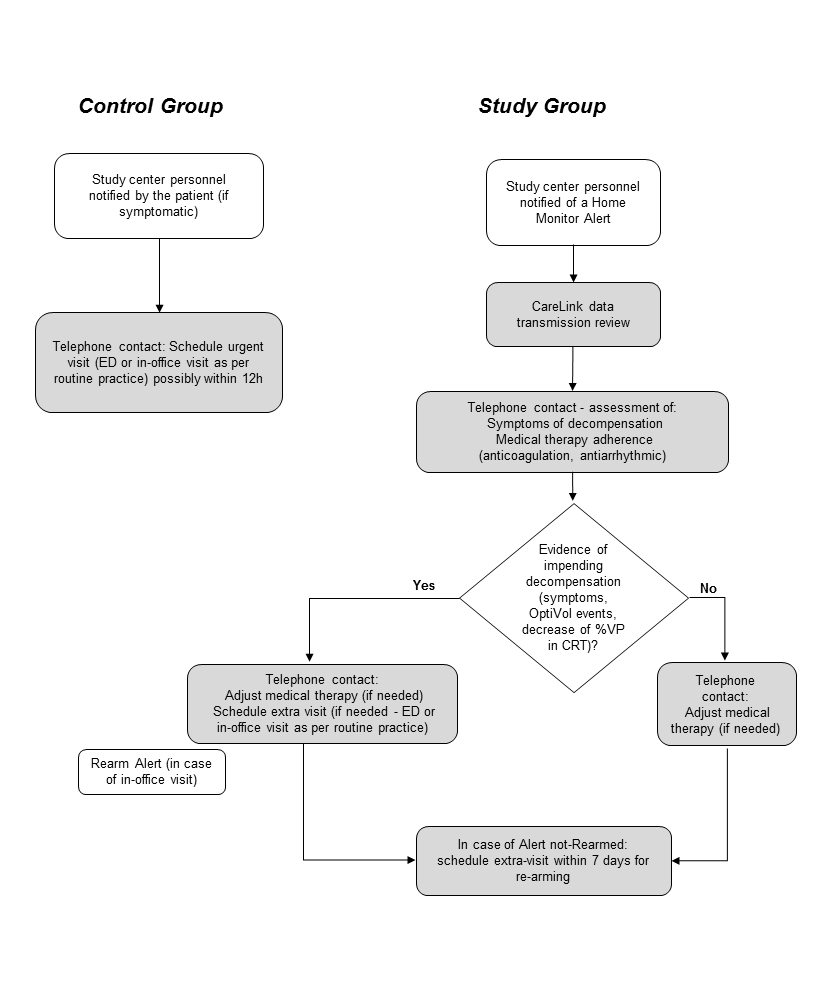

Supplement: Supplementary file 6 [file jmir_v15i8e167_app6.png]

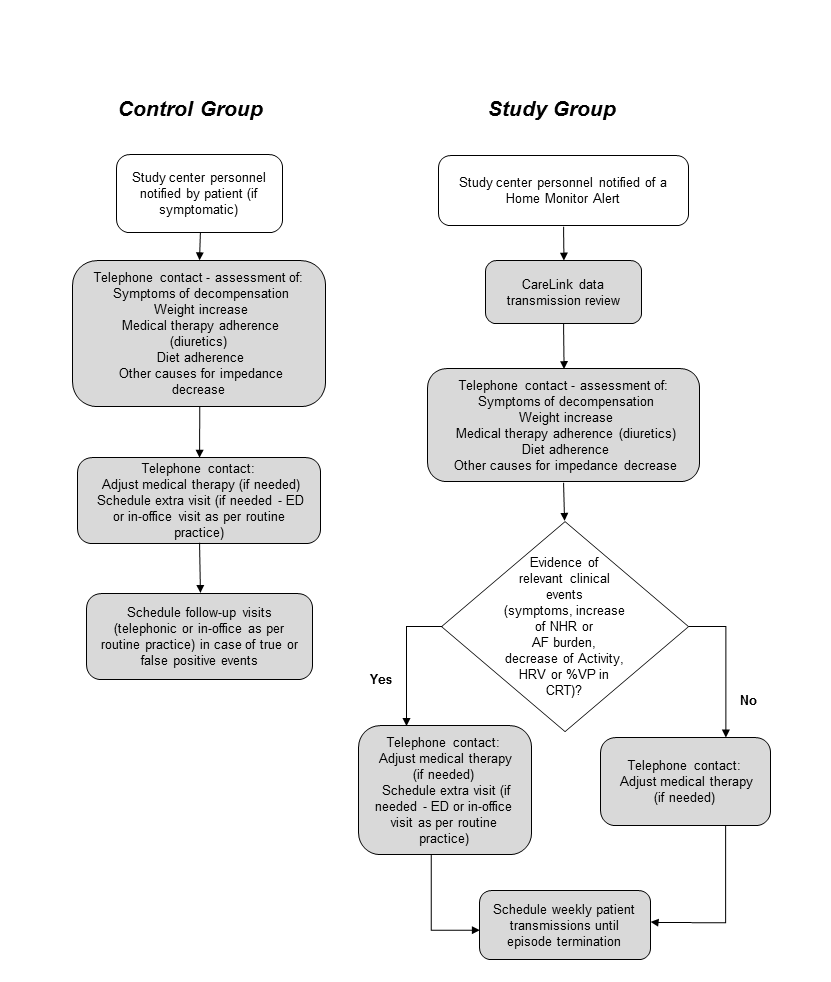

Supplement: Supplementary file 7 [file jmir_v15i8e167_app7.png]

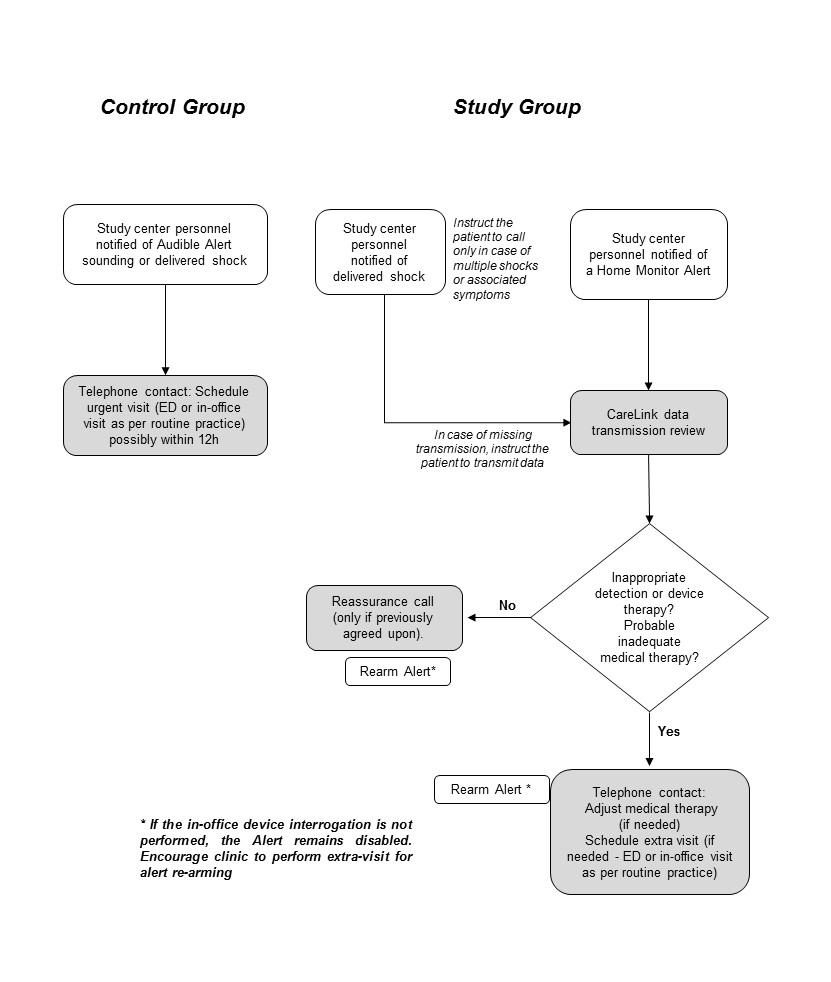

Supplement: Supplementary file 8 [file jmir_v15i8e167_app8.png]
