# Supplementary material for: The MOnitoring Resynchronization dEvices and CARdiac patiEnts (MORE-CARE) Randomized Controlled Trial: Phase 1 Results on Dynamics of Early Intervention With Remote Monitoring
Source: J Med Internet Res. 2013 Aug 21;15(8):e167. doi: 10.2196/jmir.2608 (PMC3758044; doi:10.2196/jmir.2608)
Supplement: Supplementary file 9 [file jmir_v15i8e167_app9.pdf]

CONSORT-EHEALTH Checklist V1.6.2 Report

(based on CONSORT-EHEALTH V1.6), available at [http://tinyurl.com/consort-ehealth-v1-6].

2608

Date completed

3/14/2013 6:17:09

by

Giuseppe Boriani

The MOnitoring Resynchronization dEvices and CARdiac patiEnts (MORE-CARE) Randomized Controlled trial: phase 1 results on dynamics of early intervention with remote monitoring

TITLE

1a-i) Identify the mode of delivery in the title

Yes. "remote monitoring"

1a-ii) Non-web-based components or important co-interventions in title

1a-iii) Primary condition or target group in the title

Yes. "Resynchronization dEvices and CARdiac patiEnts"

ABSTRACT

1b-i) Key features/functionalities/components of the intervention and comparator in the METHODS section of the ABSTRACT

Yes. "In this multicenter randomized controlled trial, patients with moderate to severe heart failure implanted with CRT-D devices were randomized to a Remote group (with remote follow-up and wireless automatic alerts) or to a Control group (with standard follow-up without alerts). The primary end-point of phase 1 (presented here) was the delay between an alert event and clinical decisions related to the event in the first 154 enrolled patients followed for 1 year."

1b-ii) Level of human involvement in the METHODS section of the ABSTRACT

1b-iii) Open vs. closed, web-based (self-assessment) vs. face-to-face assessments in the METHODS section of the ABSTRACT

1b-iv) RESULTS section in abstract must contain use data

1b-v) CONCLUSIONS/DISCUSSION in abstract for negative trials

INTRODUCTION

2a-i) Problem and the type of system/solution

Yes. "Modern Cardiac resynchronization therapy defibrillators (CRT-D) are equipped with reliable diagnostics able to provide a series of alerts, including lung fluid accumulation [1], occurrence of atrial fibrillation (AF) [2], or technical issues. Early diagnosis and intervention may play a crucial role in minimizing major cardiovascular events and reducing hospitalization. Several major device manufacturers offer remote monitoring (RM) capabilities [3, 4] with the aim of reducing regular follow-up visits [5] and unnecessary interim visits, or of dealing with the more complex perspective of disease management [6]. RM allows physicians to remotely access patient data and to be notified of clinical events by means of the automatic transmission of alert messages."

2a-ii) Scientific background, rationale: What is known about the (type of) system

Yes. "Previous trials such as TRUST [7] and CONNECT [8] have shown that RM is safe and reduces delay in detection of events such as AF. However, these trials either excluded [7] or included only a minority of patients with biventricular defibrillators (CRT-D) [8], and did not include an alert on lung fluid accumulation which is potentially useful in the context of heart failure management. In addition, these trials [7- 9] were not strictly focused on NYHA III-IV heart failure patients, a setting where reduction of morbidity and access to hospital may have a great significance for both the patient and the healthcare system."

METHODS

3a) CONSORT: Description of trial design (such as parallel, factorial) including allocation ratio

Yes. "MORE-CARE is a multi-center randomized trial conducted in Europe and designed in two phases [10]. Phase 1 was aimed at evaluating whether RM of CRT-D patients could shorten the time from onset of a clinically relevant event to clinical decisions in comparison with standard management (scheduled in-office visits only). The second phase of MORE-CARE is currently ongoing and is targeted at assessing whether clinical decision-making guided by RM exerts a positive impact on patient outcome (death from any cause, cardiovascular and device-related hospitalization) in comparison to standard care [10]. This manuscript is devoted to present the results of phase 1 of the study."

3b) CONSORT: Important changes to methods after trial commencement (such as eligibility criteria), with reasons

Yes. No important changes to methods occurred after trial commencement.

3b-i) Bug fixes, Downtimes, Content Changes

4a) CONSORT: Eligibility criteria for participants

Yes. "The trial design has been reported in detail elsewhere [10], the flow chart is shown in Figure 1. In summary, patients in sinus rhythm with de novo implantation of CRT-D for systolic heart failure with NYHA class III/IV symptoms and an LVEF <35% were randomized 1:1 to wireless RM (Remote group) or to a Control group."

10.Burri H, Quesada A, Ricci RP, et al. The MOnitoring Resynchronization dEvices and CARdiac patiEnts (MORE-CARE) study: Rationale and design. Am Heart J 2010; 160:42-8.

4a-i) Computer / Internet literacy

4a-ii) Open vs. closed, web-based vs. face-to-face assessments:

Yes. "The trial design has been reported in detail elsewhere [10], the flow chart is shown in Figure 1."

10. Burri H, Quesada A, Ricci RP, et al. The MOnitoring Resynchronization dEvices and CARdiac patiEnts (MORE-CARE) study: Rationale and design. Am Heart J 2010; 160:42-8.

4a-iii) Information giving during recruitment

4b) CONSORT: Settings and locations where the data were collected

Yes. "The trial design has been reported in detail elsewhere [10], the flow chart is shown in Figure 1."

10. Burri H, Quesada A, Ricci RP, et al. The MOnitoring Resynchronization dEvices and CARdiac patiEnts (MORE-CARE) study: Rationale and design. Am Heart J 2010; 160:42-8.

4b-i) Report if outcomes were (self-)assessed through online questionnaires

Yes. Outcomes were not based on self-assessed through online questionnaires. Outcomes were based on the analysis of data collected and reported by Principal Investigators on an Electronic Data Capture system.

"The trial design has been reported in detail elsewhere [10], the flow chart is shown in Figure 1."

10. Burri H, Quesada A, Ricci RP, et al. The MOnitoring Resynchronization dEvices and CARdiac patiEnts (MORE-CARE) study: Rationale and design. Am Heart J 2010; 160:42-8.

4b-ii) Report how institutional affiliations are displayed

5) CONSORT: Describe the interventions for each group with sufficient details to allow replication, including how and when they were actually administered

5-i) Mention names, credential, affiliations of the developers, sponsors, and owners

5-ii) Describe the history/development process

5-iii) Revisions and updating

5-iv) Quality assurance methods

5-v) Ensure replicability by publishing the source code, and/or providing screenshots/screen-capture video, and/or providing flowcharts of the algorithms used

5-vi) Digital preservation

5-vii) Access

Yes. "The trial design has been reported in detail elsewhere [10], the flow chart is shown in Figure 1."

10. Burri H, Quesada A, Ricci RP, et al. The MOnitoring Resynchronization dEvices and CARdiac patiEnts (MORE-CARE) study: Rationale and design. Am Heart J 2010; 160:42-8.

5-viii) Mode of delivery, features/functionalities/components of the intervention and comparator, and the theoretical framework

Yes. "In the Remote group, Automatic RM alerts for lung fluid accumulation (OptiVol), atrial tachyarrhythmia (AT/AF), lead and device integrity or inactivated VF detection/therapy were turned on at baseline using standardized predefined thresholds, which could later be modified at the physicians' discretion. All audible alerts were disabled with the exception of those related to lead and device integrity and programming.
Due to technological aspects of the RM system, any device-detected event for AT/AF, fast ventricular rate during AT/AF and shock, triggers a remote notification of the episode and automatically disables the corresponding alert which may be re-enabled only by means of an in-office device check (alert re-arming). For patients in the Control group, only audible alerts for system integrity and programming issues were enabled."

10. Burri H, Quesada A, Ricci RP, et al. The MOnitoring Resynchronization dEvices and CARdiac patiEnts (MORE-CARE) study: Rationale and design. Am Heart J 2010; 160:42-8.

5-ix) Describe use parameters

5-x) Clarify the level of human involvement

5-xi) Report any prompts/reminders used

Yes. "The trial design has been reported in detail elsewhere [10], the flow chart is shown in Figure 1."

10. Burri H, Quesada A, Ricci RP, et al. The MOnitoring Resynchronization dEvices and CARdiac patiEnts (MORE-CARE) study: Rationale and design. Am Heart J 2010; 160:42-8.

5-xii) Describe any co-interventions (incl. training/support)

Yes. ""The trial design has been reported in detail elsewhere [10], the flow
chart is shown in Figure 1."

10. Burri H, Quesada A, Ricci RP, et al. The MOnitoring
Resynchronization dEvices and CARdiac patiEnts (MORE-CARE) study:
Rationale and design. Am Heart J 2010; 160:42-8.

"For appropriate clinical decisions and patient management, physicians were aided by specific flow-charts suggesting clinical actions for each type of alert (system performance alerts, device shocks, AF “rhythm control” strategy, AF “rate control” strategy, and OptiVol events)."

6a) CONSORT: Completely defined pre-specified primary and secondary outcome measures, including how and when they were assessed

Yes."The trial design has been reported in detail elsewhere [10], the flow
chart is shown in Figure 1."

10. Burri H, Quesada A, Ricci RP, et al. The MOnitoring
Resynchronization dEvices and CARdiac patiEnts (MORE-CARE) study:
Rationale and design. Am Heart J 2010; 160:42-8.

"The primary endpoint of phase 1 was to demonstrate a reduction of the time from onset of an actionable device-detected event to a clinical decision. A clinical decision was defined by at least one of the following: change in drug therapy, device reprogramming, patient education (specific advice on salt, fluid check, exercise, behavior, etc.) as well as planning of hospital admission for other interventions (e.g., electrical cardioversion, radiofrequency ablation, etc.)."

"The primary end-point was defined as the delay between onset of the actionable device-detected event to a clinical decision related to that event."

"Phase 1 included as secondary objectives an exploratory analysis on the impact of RM on quality of life (by means of the Minnesota Living with Heart Failure Questionnaire) and clinical status (measured by the Clinical Composite Score)."

6a-i) Online questionnaires: describe if they were validated for online use and apply CHERRIES items to describe how the questionnaires were designed/deployed

6a-ii) Describe whether and how “use” (including intensity of use/dosage) was defined/measured/monitored

6a-iii) Describe whether, how, and when qualitative feedback from participants was obtained

6b) CONSORT: Any changes to trial outcomes after the trial commenced, with reasons

Yes. No changes to trial outcomes occurred after the trial commenced.

7a) CONSORT: How sample size was determined

7a-i) Describe whether and how expected attrition was taken into account when calculating the sample size

7b) CONSORT: When applicable, explanation of any interim analyses and stopping guidelines

Yes. "The trial design has been reported in detail elsewhere [10], the flow
chart is shown in Figure 1."

10. Burri H, Quesada A, Ricci RP, et al. The MOnitoring
Resynchronization dEvices and CARdiac patiEnts (MORE-CARE) study:
Rationale and design. Am Heart J 2010; 160:42-8.

8a) CONSORT: Method used to generate the random allocation sequence

Yes. For each centre, randomization was automatically and dynamically generated by the electronic data capture system usde to collect patients data.

"The trial design has been reported in detail elsewhere [10], the flow
chart is shown in Figure 1."

10. Burri H, Quesada A, Ricci RP, et al. The MOnitoring
Resynchronization dEvices and CARdiac patiEnts (MORE-CARE) study:
Rationale and design. Am Heart J 2010; 160:42-8.

8b) CONSORT: Type of randomisation; details of any restriction (such as blocking and block size)

Yes. Patients were randomized 1:1 into 2 groups at baseline visit. Randomization was stratified by center.

"The trial design has been reported in detail elsewhere [10], the flow
chart is shown in Figure 1."

10. Burri H, Quesada A, Ricci RP, et al. The MOnitoring
Resynchronization dEvices and CARdiac patiEnts (MORE-CARE) study:
Rationale and design. Am Heart J 2010; 160:42-8.

9) CONSORT: Mechanism used to implement the random allocation sequence (such as sequentially numbered containers), describing any steps taken to conceal the sequence until interventions were assigned

"The trial design has been reported in detail elsewhere [10], the flow
chart is shown in Figure 1."

10. Burri H, Quesada A, Ricci RP, et al. The MOnitoring
Resynchronization dEvices and CARdiac patiEnts (MORE-CARE) study:
Rationale and design. Am Heart J 2010; 160:42-8.

10) CONSORT: Who generated the random allocation sequence, who enrolled participants, and who assigned participants to interventions

Yes. Participants were enrolled by Invetsigators of involved centres after evaluating that all inclusion/exclusion criteria were met. Each Patient was randomized in a 1:1 fashion into one of two study arms. Randomization was automatically and dynamically generated by the electronic data capture system usde to collect patients data.

"The trial design has been reported in detail elsewhere [10], the flow
chart is shown in Figure 1."

10. Burri H, Quesada A, Ricci RP, et al. The MOnitoring
Resynchronization dEvices and CARdiac patiEnts (MORE-CARE) study:
Rationale and design. Am Heart J 2010; 160:42-8.

11a) CONSORT: Blinding - If done, who was blinded after assignment to interventions (for example, participants, care providers, those assessing outcomes) and how

11a-i) Specify who was blinded, and who wasn't

Yes. "The trial design has been reported in detail elsewhere [10], the flow
chart is shown in Figure 1."

10. Burri H, Quesada A, Ricci RP, et al. The MOnitoring
Resynchronization dEvices and CARdiac patiEnts (MORE-CARE) study:
Rationale and design. Am Heart J 2010; 160:42-8.

11a-ii) Discuss e.g., whether participants knew which intervention was the "intervention of interest" and which one was the "comparator"

11b) CONSORT: If relevant, description of the similarity of interventions

Not relevant for this study.
12a)

12a) CONSORT: Statistical methods used to compare groups for primary and secondary outcomes

In accordance with the sample size estimation reported in the study design [10], the sample size requirement was 77 subjects per arm. In the present report of phase 1 results, data analysis was performed according to the intention-to-treat principle. The analysis for the primary end-point includes all randomized subjects who experienced at least one event. Similarly to a previous study [8], if a patient experienced multiple events of a specific type (e. g., ≥6 h AT/AF burdens in a day) between 2 consecutive evaluations, only the first of these was paired with the next device interrogation/visit and counted toward the analysis. For every patient, an average time from event onset to clinical decision was calculated for each type of event and entered in the analysis per event type.

Descriptive statistics are reported as mean ± 1 standard deviation for normally distributed continuous variables, or medians with 25th to 75th percentiles for skewed variables. Normality of distribution was tested by the Kolmogorov-Smirnov test. Absolute and relative frequencies are reported for categorical variables. Continuous Gaussian variables were compared by the Student's t-test for independent samples, while skewed distributions were compared using the Mann-Whitney non-parametric test. Differences in proportions were compared by applying Chi-square analysis. Rates were compared by means of the Comparison Incidence Rates (Large Sample) Test. An alpha-level of 0.05 was considered for each test. All statistical analyses were performed by using SAS 9.3 version software (SAS Institute Inc., Cary, NC, USA).

12a-i) Imputation techniques to deal with attrition / missing values

Yes. "The final patient cohort object of analysis was comprised of 148 patients (76 in the Remote group and 72 in the Control group, see Figure 2)."

"Figure 2. Phase 1 follow-up experience flow-chart."

12b) CONSORT: Methods for additional analyses, such as subgroup analyses and adjusted analyses

No subgroups analyses were conducted.

RESULTS

13a) CONSORT: For each group, the numbers of participants who were randomly assigned, received intended treatment, and were analysed for the primary outcome

Yes. "A total of 154 patients were enrolled from May 2009 through April 2010 from 32 centers in 6 different countries (France, Hungary, Israel, Italy, Spain and Switzerland). The final patient cohort object of analysis was comprised of 148 patients (76 in the Remote group and 72 in the Control group, see Figure 2)."

"Figure 2. Phase 1 follow-up experience flow-chart."

13b) CONSORT: For each group, losses and exclusions after randomisation, together with reasons

Yes. "The final patient cohort object of analysis was comprised of 148 patients (76 in the Remote group and 72 in the Control group, see Figure 2)."

"Figure 2. Phase 1 follow-up experience flow-chart."

13b-i) Attrition diagram

14a) CONSORT: Dates defining the periods of recruitment and follow-up

Yes. "A total of 154 patients were enrolled from May 2009 through April 2010 from 32 centers in 6 different countries (France, Hungary, Israel, Italy, Spain and Switzerland)."

14a-i) Indicate if critical "secular events" fell into the study period

14b) CONSORT: Why the trial ended or was stopped (early)

The trial did not end or was stopped early.

15) CONSORT: A table showing baseline demographic and clinical characteristics for each group

Yes. "Demographic data and clinical parameters of the population under analysis were similar between the study arms at the time of enrollment (Table 1)."

15-i) Report demographics associated with digital divide issues

Yes. "Demographic data and clinical parameters of the population under analysis were similar between the study arms at the time of enrollment (Table 1)."

16a) CONSORT: For each group, number of participants (denominator) included in each analysis and whether the analysis was by original assigned groups

16-i) Report multiple "denominators" and provide definitions

Yes.

16-ii) Primary analysis should be intent-to-treat

17a) CONSORT: For each primary and secondary outcome, results for each group, and the estimated effect size and its precision (such as 95% confidence interval)
Yes.
17a-i) Presentation of process outcomes such as metrics of use and intensity of use

17b) CONSORT: For binary outcomes, presentation of both absolute and relative effect sizes is recommended
Yes.
18) CONSORT: Results of any other analyses performed, including subgroup analyses and adjusted analyses, distinguishing pre-specified from exploratory
No other analyses were performed.
18-i) Subgroup analysis of comparing only users

19) CONSORT: All important harms or unintended effects in each group
Yes. No important harms or unintended effects were encountered for both groups.
19-i) Include privacy breaches, technical problems

19-ii) Include qualitative feedback from participants or observations from staff/researchers

DISCUSSION

20) CONSORT: Trial limitations, addressing sources of potential bias, imprecision, multiplicity of analyses
20-i) Typical limitations in ehealth trials
Yes. "Phase 1 of MORE-CARE was not powered for evaluating the impact of RM on hospitalizations and mortality, which is being studied in the second phase of the trial. There were only a few cases of system integrity alerts because of the limited 1-year follow-up. These aspects will be better evaluated with the longer observation period of the ongoing trial."
21) CONSORT: Generalisability (external validity, applicability) of the trial findings
21-i) Generalizability to other populations

21-ii) Discuss if there were elements in the RCT that would be different in a routine application setting

22) CONSORT: Interpretation consistent with results, balancing benefits and harms, and considering other relevant evidence
22-i) Restate study questions and summarize the answers suggested by the data, starting with primary outcomes and process outcomes (use)
Yes. "The MORE-CARE trial is the first European large-scale randomized study evaluating disease management guided by RM (including lung fluid overload alerts) in a population comprised exclusively of CRT-D patients with advanced heart failure. The main finding of phase 1 of the study that we report here is that a wireless RM strategy permits physicians to take clinical decisions 27 days sooner as compared to standard in-office care, while reducing the total number of in-hospital visits.
Several recent studies [7-9, 11-14] have demonstrated the benefits associated with remote patient monitoring in terms of early detection of relevant events as well as reduction of in-office follow-up visits. However, none of these studies was dedicated specifically to CRT-D patients with advanced heart failure and were mostly conducted in the United States. Recently, the results of the EVOLVO trial were reported, showing a significant reduction in emergency visits in patients on RM [9]. The EVOLVO trial, however, differed significantly from MORE-CARE in that the patient population was enrolled exclusively in one region of Italy, with a mixture of ICD and CRT-D patients of whom >80% had NYHA class I/II heart failure, with activation of audible alerts in the control arm and without evaluation of delay in medical decisions [9, 15]."
22-ii) Highlight unanswered new questions, suggest future research

Other information

23) CONSORT: Registration number and name of trial registry
Yes. "Clinical Trial Registration: URL: http://clinicaltrials.gov/ Identifier: NCT00885677"
24) CONSORT: Where the full trial protocol can be accessed, if available
Yes. "Clinical Trial Registration: URL: http://clinicaltrials.gov/ Identifier: NCT00885677"

10. Burri H, Quesada A, Ricci RP, et al. The MOnitoring
Resynchronization dEvices and CARdiac patiEnts (MORE-CARE) study:
Rationale and design. Am Heart J 2010; 160:42-8.

25) CONSORT: Sources of funding and other support (such as supply of drugs), role of funders
Yes. "The MORE-CARE study is funded by Medtronic, Inc (Minneapolis, MN)."
X26-i) Comment on ethics committee approval

x26-ii) Outline informed consent procedures

X26-iii) Safety and security procedures

X27-i) State the relation of the study team towards the system being evaluated
